# 100 km wear-free sliding achieved by microscale superlubric graphite/DLC heterojunctions under ambient conditions

**DOI:** 10.1093/nsr/nwab109

**Published:** 2021-06-24

**Authors:** Deli Peng, Jin Wang, Haiyang Jiang, Shuji Zhao, Zhanghui Wu, Kaiwen Tian, Ming Ma, Quanshui Zheng

**Affiliations:** Department of Engineering Mechanics, Tsinghua University, Beijing 100084, China; Center for Nano and Micro Mechanics, Tsinghua University, Beijing 100084, China; Institute of Superlubricity Technology, Research Institute of Tsinghua University in Shenzhen, Shenzhen 518057, China; Department of Engineering Mechanics, Tsinghua University, Beijing 100084, China; Center for Nano and Micro Mechanics, Tsinghua University, Beijing 100084, China; Institute of Superlubricity Technology, Research Institute of Tsinghua University in Shenzhen, Shenzhen 518057, China; Center for Nano and Micro Mechanics, Tsinghua University, Beijing 100084, China; State Key Laboratory of Tribology, Tsinghua University, Beijing 100084, China; Department of Engineering Mechanics, Tsinghua University, Beijing 100084, China; Center for Nano and Micro Mechanics, Tsinghua University, Beijing 100084, China; Institute of Superlubricity Technology, Research Institute of Tsinghua University in Shenzhen, Shenzhen 518057, China; Center for Nano and Micro Mechanics, Tsinghua University, Beijing 100084, China; Institute of Superlubricity Technology, Research Institute of Tsinghua University in Shenzhen, Shenzhen 518057, China; State Key Laboratory of Tribology, Tsinghua University, Beijing 100084, China; Department of Mechanical Engineering, Tsinghua University, Beijing 100084, China; Department of Engineering Mechanics, Tsinghua University, Beijing 100084, China; Center for Nano and Micro Mechanics, Tsinghua University, Beijing 100084, China; Institute of Superlubricity Technology, Research Institute of Tsinghua University in Shenzhen, Shenzhen 518057, China; State Key Laboratory of Tribology, Tsinghua University, Beijing 100084, China

**Keywords:** structural superlubricity, wear-free, microscale, graphite, DLC

## Abstract

Wear-free sliding between two contacted solid surfaces is the ultimate goal in the effort to extend the lifetime of mechanical devices, especially when it comes to inventing new types of micro-electromechanical systems where wear is often a major obstacle. Here we report experimental observations of wear-free sliding for a micrometer-sized graphite flake on a diamond-like-carbon (DLC) surface under ambient conditions with speeds up to 2.5 m/s, and over a distance of 100 km. The coefficient of friction (COF) between the microscale graphite flake, a van der Waals (vdW) layered material and DLC, a non-vdW-layered material, is measured to be of the order of ${10^{ - 3}}$, which belongs to the superlubric regime. Such ultra-low COFs are also demonstrated for a microscale graphite flake sliding on six other kinds of non-vdW-layered materials with sub-nanometer roughness. With a synergistic analysis approach, we reveal the underlying mechanism to be the combination of interfacial vdW interaction, atomic-smooth interfaces and the low normal stiffness of the graphite flake. These features guarantee a persistent full contact of the interface with weak interaction, which contributes to the ultra-low COFs. Together with the extremely high in-plane strength of graphene, wear-free sliding is achieved. Our results broaden the scope of superlubricity and promote its wider application in the future.

## Teaser

Wear-free sliding for an ultra-long distance of 100 km is achieved by microscale superlubric graphite/DLC heterojunctions under ambient conditions, promising wide application in micro-electromechanical systems, hard drives, bearings etc.

## INTRODUCTION

Although the first microscale rotary electrostatic side drive motor (micro-motor) was demonstrated in 1988 [1], the lifetimes of all micro-motors reported so far are too short for real application due to severe wear, which is significant at this scale because of the large surface-to-volume ratio [2,3]. By operating in a nitrogen environment, the lifetime of micro-motors with polysilicon/polysilicon contacts was significantly extended to millions of cycles over a period of several days [4,5]. However, they are still far from becoming commercial products. Under such small confinements, traditional tribological and lubrication methods used at the macroscale are ineffective [2,3,6]. Therefore, in order to avoid rubbing parts, most commercial micro-electromechanical systems (MEMSs) used today, such as inertial sensors [7], optical MEMSs [8] and radio frequency switches [9], are designed to move through bending or twisting of a beam. That is, using the deformation of a material to achieve movement, instead of sliding or rotating parts like in the macroscopic world. However, only limited types of movement can be achieved in this way, and the displacement is usually very small [10]. These severely limit the advances in MEMSs [6].

Structural superlubricity (SSL), a state of nearly vanishing friction and no wear between two solid surfaces in contact [3,11–14], provides a revolutionary solution to this challenge. Since the contact area of moving parts within MEMSs are all at the microscale, the realization of microscale contacts of SSL in 2012 [15] opened a door to the exploration and design of brand new kinds of MEMSs [3], such as SSL nanogenerators (SLNGs) [16,17] and SSL resonators [18]. The former generates electricity by sliding a charged electrode across the superlubric interfaces, causing a variation in capacitance. With their lifetime greatly increased, SLNGs are also theoretically proven to possess superb performance—three orders of magnitude enhancement in current densities and output powers compared with conventional nanogenerators [16].

The characteristic frequency of MEMSs’ working parts is usually at or below MHz [19,20], and the corresponding displacement is often smaller than one micrometer [19,20]. Thus, their maximum working velocity is on or below the order of meter per second. As MEMSs are mainly used to transfer mechanical and/or electrical signals or convert between them, they are rarely required to work under high pressure [6,21]. Under these typical working conditions of MEMSs, SSL has been experimentally shown to achieve extremely low friction [18,22,23]. While it is reasonable to anticipate wearless sliding enabled by SSL, which is critical for the successful design of MEMSs with moving parts, no experimental observation based on SSL has been reported. Using highly hydrogenated diamond-like-carbon (DLC) films, a coefficient of friction (COF) as small as 0.001 to 0.005 has been achieved, and very low wear rates of 10^−11^ to 10^−10^ mm^3^/(N · m) were demonstrated [24]. After a running-in process under ambient conditions, superlubricity was achieved using 2D heterostructures such as graphene/MoS_2_, and a COF of 10^–3^ magnitude could be maintained over 10^6^ loops under a nitrogen environment [25]. However, these good tribological experimental results are limited to either inert-gas or high-vacuum environments [24,25]. Experimental reports on wear-free solid contact sliding under ambient conditions are still lacking [3].

Here we show experimental observation of over 100 km of wear-free sliding at a speed of 2.5 m/s with a load of ∼10 μN under ambient conditions. This is achieved by constructing direct contact between a microscale graphite flake and a macroscale DLC surface. Our experimental measurements show that the COF of this system is of the order of 0.001. Such a low COF is also found with the microscale graphite flake and the other six kinds of non-van der Waals (vdW)-layered materials with sub-nanometer roughness. Using continuum mechanical analysis and the finite element method, we reveal that the full contact at the interface, extremely high in-plane strength of graphene and weak vdW interaction across the interface altogether ensure the observed superlubricity and extremely high wear resistance.

## RESULTS AND DISCUSSION

**Figure 1.**
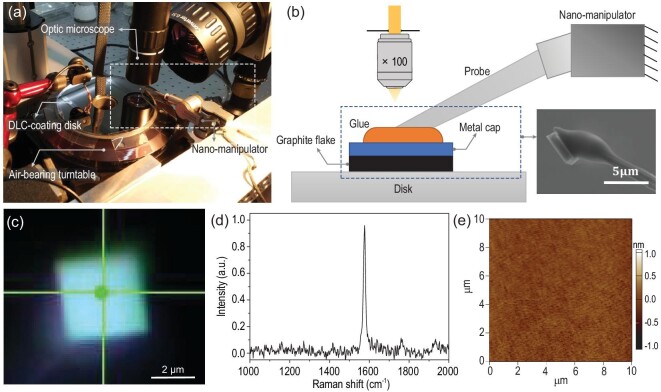
100 km sliding test between the graphite flake and DLC. (a) Experiment set-up. (b) Illustration of the experimental set-up for the part highlighted by the white dotted box in (a). The inset picture shows the SEM picture of the probe-flake sample. (c) Optical image of the bottom surface of the graphite flake, which was in contact with DLC after sliding. (d) Raman spectrum at the location of the green point in (c). (e) AFM morphology image on the sliding track of DLC after sliding.

A home-built micro wear test set-up was used to perform long-distance sliding experiments, as shown in Fig. 1a, and illustrated in Fig. 1b. A 4-inch DLC-coated disk was fixed on a high-precision air-bearing turntable (ABRT-150, Aerotech). A graphite flake with a 100-nm-thick metal cap (50-nm-thick Au and 50-nm-thick Al) was glued to the tip of a tungsten probe (see scanning electron microscope (SEM) picture in the inset picture of Fig. 1b). This flake was sheared from the top of the microscale square graphite mesa (4 μm × 4 μm) showing self-retraction motion (SRM) [15,26]. The mesas were made by lithography from highly oriented pyrolytic graphite (HOPG) [15]. The bottom surface of the graphite flake, i.e. the sheared upper part of the graphite mesa, is single crystalline, atomically smooth and has almost no defects [26,27]. The root of the tungsten probe was fixed on a piezo-driven nano-manipulator (Kleindiek MM3A) to apply a normal load on the graphite flake, which was ∼10 μN. The calibration of this normal load is detailed in Supplementary Section 6. The graphite flake was pressed against the disk, which was anchored on the turntable. The rotation of the turntable would cause the graphite flake and the disk to slide relative to each other. In our experiment, the angular velocity of the turntable was set as 1000 rpm (revolutions per minute), and the radial distance between the graphite flake and the center of the disk was ∼2.4 cm, which resulted in a sliding speed between the flake and disk of ∼2.5 m/s. As illustrated in Fig. 1b, a 100× optical lens (HIROX RH-8800) was used to monitor the motion of the graphite flake during the sliding process *in situ*. The whole wear test was performed in a class-1000 clean room.

After 12 hours of sliding at a constant speed of 2.5 m/s, which amounts to a sliding distance of 108 km, we characterized the bottom surface of graphite with Raman spectra (Horiba Evolution) by turning over the graphite flake. As shown in Fig. 1c and d, the absence of D peak around the center of the graphite flake indicated a wearless sliding process. The Raman spectra around the four corners of this flake also showed no D peak (details in Supplementary Section 4). We further checked the morphology of the sliding track on the DLC surface using the optical microscope and atomic force microscope (AFM) after the sliding, and no wear track or wear debris was observed (Fig. 1e). These measurements indicated no wear on either of the surfaces.

**Figure 2.**
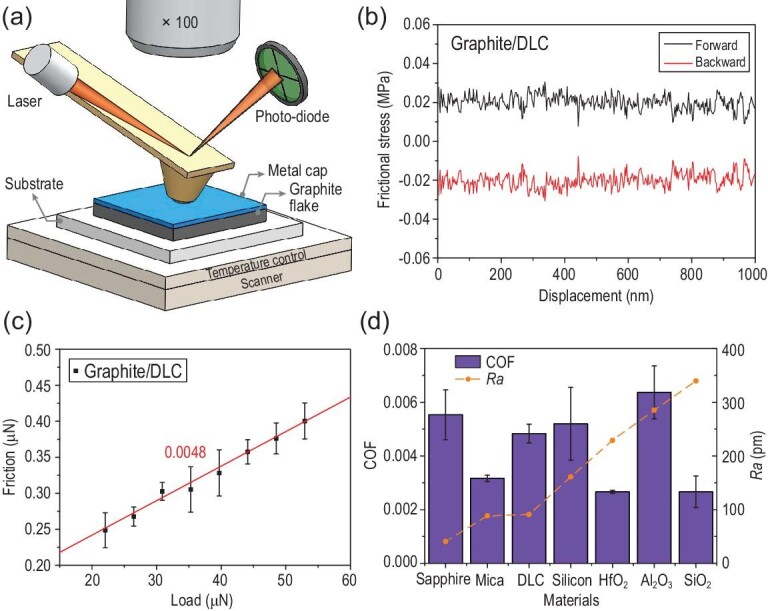
Experimental set-up for friction measurements. (a) Schematic diagram of the experimental set-up to measure the friction between the graphite flake and different substrates. The 100× objective lens was coupled to the AFM heads to observe the motion between the graphite and the substrate *in situ*. (b) Friction loop between the graphite flake and DLC. The title of the abscissa axis, displacement, refers to the relative sliding displacement between the graphite flake and DLC. (c) COFs between graphite flake and DLC. The friction force can be written as *F*_f_ = *μF*_N _+ *F*_0_, where *F*_f_ is friction force, *μ* represents the COF, *F*_N_ is normal force and *F*_0_ is the offset friction force when *F*_N _= 0. By fitting the slope between friction and normal force, we obtained the COF between the flake and DLC: 0.0048 with a standard error of 0.0003. (d) Results of the COFs between the graphite flake and seven kinds of non-vdW-layered materials, and arithmetic mean roughness (*Ra*) values of the materials’ surfaces. These experiments were conducted under ambient conditions (temperature: 20–25°C; relative humidity: 20%–30%). During friction measurements, the scan velocity was kept constant at 1500 nm/s. *Ra* values were calculated from the morphology of the substrate's surfaces within a 1 μm × 1 μm square area measured with AFM. The COF error bars in (d) represent the standard deviation of the results obtained from three graphite flake samples.

The experimental result of realizing 100 km of wear-free sliding is surprising. To understand the mechanism underlying the extraordinary anti-wear property of this system, we measured the corresponding COF as illustrated in Fig. 2a. For the friction measurement under ambient conditions, the experiment set-up included a commercial atomic force microscope (NT-MDT, Russia), a 100 μm *XYZ* piezoelectric tube scanner and a 100× optical objective, which could provide real-time observation as the probe manipulated the graphite samples. The substrate was fixed on the scanner, and the flake covered by metal cap was pressed with a special visible AFM tip (VIT_P AFM probe, NT-MDT, Russia). The nominal stiffness of the cantilever was ∼50 N/m. When the flake was held by the AFM tip, horizontal displacement of the scanner caused shear motion between the flake and the substrate. Thus, the normal force applied by the tip could be precisely controlled and shear force could be measured simultaneously in real time [26]. The normal load was controlled between 20 and 60 μN. Figure 2b presents the friction loop between the graphite flake and DLC. The closed area by the forward and backward curves represents the frictional energy dissipation. The average frictional stress (the average amount of friction per unit area across the sliding interface) is extremely low [3,28,29], ∼0.02 MPa for 40 μN normal load. Figure 2c shows the typical dependence of friction on normal force between the graphite flake and DLC. The COF is estimated to be 0.005, which falls in the regime of superlubricity. The average friction force increases linearly with the normal load. Notably, by extrapolating the linear fitting line to the point corresponding to zero normal load, a finite friction of 0.22 μN could be estimated, which is probably due to the adhesion between the graphite flake and DLC. This finite friction with zero normal load was also observed in the previous work [30], where a graphite-coated microsphere slid on SiO_2_ and graphite substrates.

Due to the π electrons within graphene, the interaction between the surfaces of graphite flakes and DLC is mainly weak vdW interaction, which could be one reason for such low frictional stress and COF. To check the generality of the ultra-low friction and wearless phenomenon, we further tested the friction properties between the graphite flake and other non-vdW-layered materials, including sapphire (α-Al_2_O_3_, (0001)), natural mica (001), silicon (111), amorphous HfO_2_ film, amorphous Al_2_O_3_ film and amorphous SiO_2_ film. The results are similar to those between the graphite flake and DLC, which are summarized in Fig. 2d and detailed in Supplementary Figs S1 and S2. The average frictional stress varies from 0.015 to 0.03 MPa (Supplementary Fig. S1). All the COFs of the seven materials are of the order of magnitude of 10^−3^_._ The smoothest material we measured was sapphire with an arithmetic mean roughness (Ra) of 0.04 nm and a corresponding COF of 0.0055. For a relatively rough material such as the silicon oxide film, the corresponding COF is 0.0026. Interestingly, for all the systems studied here, there is no obvious correlation between COF and surface roughness, which can be seen in Fig. 2d.

The observed microscale superlubricity under ambient conditions for non-vdW-layered materials, including both crystalline and amorphous materials, is beyond our expectation [3,11,14,31]. For superlubricity with single contact, i.e. a full contact where the real contact area is almost the same as the nominal contact area, various nanoscale systems have been validated in experiments [28,32,33]. However, at the microscale, the interfaces are limited to the homojunction made by graphite [15,23] or heterojunction composed of graphite and hBN, both of which belong to the vdW-layered materials in general [14,26]. For superlubricity with multi-contacts where the real contact area is much smaller than the nominal contact area, a macroscale superlubric interface was realized in recent years by making use of the numerous nanoscale single contacts as third bodies, e.g. nanodiamond particles scrolled by graphene or MoS_2_ [34,35], on substrates of non-vdW materials, e.g. DLC and SiO_2_. However, for such systems, the large stress concentration, requirement for low humidity and huge contact edge effects are difficulties still to be overcome [3]. In addition, the introduction of the third bodies may limit application where direct contact between two solid surfaces is required, e.g. electric contact. There are also other attempts to achieve superlubricity on 2D/non-vdW-layered material interfaces, such as sliding silicon lenses or DLC tips on chemical vapor deposition-growth graphene or mechanic exfoliated graphene. The COFs measured, however, are all above 0.01 [36,37]. One reason could be the imperfections within the 2D materials used, such as defects or wrinkles [11]. In our system, the graphite flake exhibiting SRM guarantees a single crystal graphene surface free of defects and wrinkles [26,27]. Another reason may be the presence of multi-contacts, which easily causes high stress concentration at those points upon first contact, and destroys 2D materials [38,39]. For the surfaces studied here, the roughness of the non-vdW-layered materials is about sub-nanometer. Thus, it is interesting to know whether multi-contacts still form in our systems.

**Figure 3.**
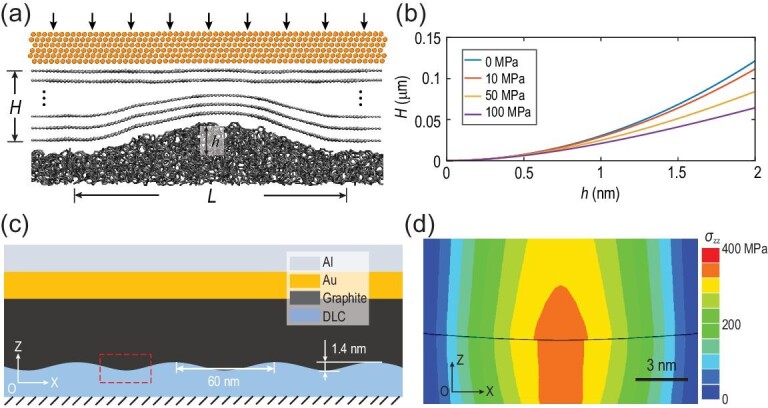
Contact between 2D materials and non-vdW-layered materials. (a) Schematic diagram of the contact between graphite with a rigid cap and rough substrates. *H* is the total thickness of the graphite, *h* is the height of the rough peak and *L* is the width of the rough peak. (b) Visualization of Equation 1 with the characteristic external pressure used in experiments. Parameters used here (Supplementary Sections 7 and 8): $\gamma \ = \ 0.2253\ {\rm{J}}/{\rm{m}}$, $E\ = \ 36.5\ {\rm{GPa}}$ and $a\ = \ 4\ {\rm{\mu m}}$. (c) Schematic diagram of the FEM model. (d) Normal stress (σ_zz_) distribution within the red box in Fig. 3c.

While direct measurements of the contact state would be most convincing, in the present study, as the thickness of the graphite flake together with the metal cap is several hundred nanometers and non-transparent, it is difficult to estimate the real contact area directly from experiments. In this case, considering the elastic deformation of the graphite flake under normal load during the friction test (illustrated in Fig. 3a), the thickness of the graphite flake should meet the following criterion to achieve full contact with the substrate (detailed in Supplementary Section 8):

(1) $$\begin
{equation}
H > \ {H_c} = \frac{{3Ea{h^2}}}{{16\left( {\gamma a + {p_{zz}}ha} \right)}}\ ,
\end{equation}$$

where *H_c_* is the critical thickness of the graphite flake above which the graphite will fully contact with the substrate, *E* is the Young's modulus of graphite along the normal direction [40], *a* is the side length of the graphite flake, *h* is the height of the rough peak, $\gamma $ is the surface energy and *p_zz_* is the external normal stress. In our experiment, the surface of SiO_2_ is the roughest among all seven surfaces (Fig. 2d). We choose the rough peak with the largest curvature and height on the SiO_2_ surfaces to consider its contact condition with the graphite, where *h *≈ 1.4 nm and *L* ≈ 60 nm (Supplementary Section 9). Based on Equation 1, in the experiments with normal stress of the order of MPa and *h = *1.4 nm, the total thickness *H* should be larger than 59.2 nm (Fig. 3b). Since *H* in our experiments ranges from 125 to 370 nm (Supplementary Section 2) and *p_zz_* is between 1 and 4 MPa, it is reasonable to assume a full contact on the interface between the graphite flake and the substrates.

We further used the finite element method (FEM) to estimate the contact condition with a more practical set-up (detailed in Supplementary Section 7). The vdW interactions between graphite and substrate, the presence of the metal cap and the external normal load were all taken into consideration explicitly. The maximum peak-to-peak value was 1.4 nm with a lateral size of 60 nm, shown in Fig. 3c, for SiO_2_ (Supplementary Section 9). Since the dispersion interaction between graphite and DLC atoms has been well documented as Lenard-Jones (LJ) potential [41], we took such potential to describe the interaction between graphite and the substrate. It should be noted that the dispersion interaction between graphite and SiO_2_ is similar [42]. Metal caps composed of 50-nm-thick Au and 50-nm-thick Al layers were considered. Using the pressure-displacement relation (Supplementary Fig. S8) derived from LJ potentials, we found that the adhesive pressure (924 MPa) caused by the vdW interaction itself was large enough to overcome the de-adhesion pressure (<400 MPa, shown in Fig. 3d), which was caused by the restoring force resulting from the bending of the graphite flake with thickness of 100 nm. As shown in Fig. 3b, it is easier for thicker graphite to form full contact with the substrate. Thus, the results of continuum mechanics and FEM together provide a clear picture that the graphite flake used in our experiment (thickness ≥125 nm) keeps full contact with the substrates.

The analysis above clearly shows that the ultra-flat contacting surfaces, as well as the low normal stiffness of the graphite flake, are essential in achieving a stable single (full) contact at the interface during sliding. This is important for achieving sustained superlubricity. First, full contact could eliminate stress concentration compared to multi-contacts. Second, full contact ensures that the real contact area does not change with the load, which can be another key factor in achieving an ultra-low COF. Last but not least, while edges could be a significant source of energy dissipation for superlubricity [43], full contact enables a minimum overall length of the contacting edges for a given nominal contact area. Interestingly, since there always exists full contact for the seven kinds of surfaces studied here, COF should also be independent of the surface roughness, and this is indeed what we observed in experiments (Fig. 2d). Thus, the merits of the systems, that is, the vdW interaction across the interface, ultra-flat surfaces and weak normal stiffness of the flake, constitute the essential conditions for achieving superlubricity. Together with the extremely high in-plane strength of graphene [44] and hardness of the non-vdW-layered materials [45], stable atomic structures of the contacting surfaces are maintained during sliding. As a result, 100 km of wear-free sliding is observed. We noticed that compared with graphite/graphite homojunction [15] and graphite/hBN heterojunction [26], the COF measured here is about ten times larger (2 × 10^−3^ to 6 × 10^−3^ shown in Fig. 2d compared with <1.4 × 10^−4 ^[26]). This is probably due to the additional energy dissipation caused by the asperities on the non-vdW-layered substrates during sliding (for detailed discussion see Supplementary Section 11).

**Figure 4.**
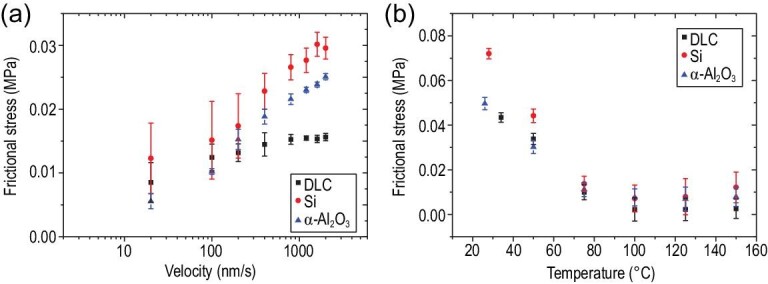
Velocity and temperature dependence of friction. (a) Velocity dependence of friction between the graphite flake and three substrates: DLC, silicon and ${\rm{\alpha }}$-Al_2_O_3_. The velocity ranges from 20 to 2000 nm/s. The normal load is 42.2 μN, and scan range is 1 μm. (b) Temperature dependence of friction between the graphite flake and the same three substrates. The normal load is 94.8 μN, and the scan range is 1 μm. The sliding velocity was kept at 1500 nm/s. The frictional stress error bars in (a) and (b) represent the standard deviation of the results obtained from 15 independent friction measurements.

The demonstrated wear-free, frictionless sliding across seven different systems opens an avenue towards practical applications of MEMSs based on superlubricity. Their robustness in real working conditions thus becomes important. Among the many interesting potential factors, we start with velocity and temperature since they are the most versatile factors across different situations. For the velocity dependence of friction, we chose DLC, silicon and ${\rm{\alpha }}$-Al_2_O_3_ as three typical materials for the substrate. Friction was measured with sliding speed from 20 nm/s to 2 ${\rm{\mu }}$m/s under ambient conditions as shown in Fig. 4a. Within the tested velocity range, all three materials showed a similar logarithmic increase of friction with velocity, which agrees with the prediction of the Prandtl-Tomlinson model [46–48], indicating the thermo-activated nature of the sliding process. Interestingly, a similar logarithmic relationship between friction and velocity was also obtained in many 2D vdW heterostructures under superlubric conditions, including contacts of graphite/graphite [49], graphite/hBN [26] and MoO_3_/MoS_2_ [50]. For temperature dependence, the substrates (DLC, silicon and ${\rm{\alpha }}$-Al_2_O_3_) were heated by a ceramic heater with a maximum temperature of 150°C. The entire heating process and friction test were carried out in a nitrogen-protected environment (relative humidity <4%) to avoid the influence of water vapor. As shown in Fig. 4b, for all the surfaces, friction shows similar dependence on temperature. Generally, friction decreases as temperature increases until 100°C. From 100°C to 150°C, however, friction hardly changes. A similar dependence of friction on temperature for a graphite flake sliding on a DLC surface has been reported [51]. Through x-ray photoelectron spectroscopy (XPS) analysis and the Langmuir adsorption model, such temperature dependence is attributed to the desorption of chemical groups from the edges of the graphite flake while temperature increases. It was also reported that increasing the temperature results in a decreasing impurity concentration on the edges of the graphite flake and thus causes significantly reduced friction [49,52]. Since the processes for fabrication and frictional tests as reported [51] are the same as present studies, we believe the mechanism for the temperature dependence of friction should also hold.

## CONCLUSION

To conclude, we have achieved 100 km of wear-free sliding between a microscale graphite flake and a macroscale DLC surface under ambient conditions. Further experiments show that the sliding between a microscale graphite flake and seven non-vdW-layered materials with sub-nanometer surface roughness, including both crystalline and amorphous materials, are superlubric under ambient conditions. All the COFs are of the order of ${10^{ - 3}}$ and are independent of the surface roughness. Through continuum mechanical analysis and FEM, we reveal the mechanism to be a synergetic effect. The atomically smooth morphology and small normal stiffness of a graphite flake enable full contact and eliminate stress concentration upon loading. Together with the weak vdW interaction across the interface, superlubricity is achieved. Further, the extremely high in-plane strength of graphene enables an extraordinary wear resistance.

Current commercial MEMS devices are designed to avoid sliding surfaces in direct contact. This excludes many exciting concepts and designs of MEMSs consisting of gears, cranks and motors for practical application [6]. With our wear-free experimental results, it would be encouraging to remove such a fundamental constraint, enabling brand-new designs of MEMSs based on sliding modes. However, how to achieve batch transfer of graphite materials, or even grow them directly on the surfaces of MEMS devices that need to contact or slide, is a major challenge to be investigated.

## METHODS

### Sample and substrate preparation

To test the friction characteristics between the graphite flake and seven non-vdW-layered materials, using a method similar to our previous articles [26,51], firstly, we dragged the graphite flake off a graphite mesa showing SRM by shearing the mesa with a probe controlled by nano-manipulator. This flake would adhere to the tip due to vdW forces. Then, we transferred it onto the target surfaces. As the vdW forces between the flake and surfaces are much stronger than the tip due to a larger contact area, the flake detached from the tip and adhered to target surfaces. Then we used the AFM in contact mode to measure the friction loop. Results are shown in Supplementary Fig. S1.

The SiO_2_ film was deposited on a silicon (111) surface using plasma-enhanced chemical vapor deposition (PECVD). The Al_2_O_3_ film and HfO_2_ film were deposited on a silicon (111) surface using atomic layer deposition (ALD). The disk coated with DLC was prepared from the disk of a hard disk drive without lubricant (manufactured by the West Digital Corporation) [51]. The surface of mica was prepared by mechanically cleaving a millimeter-sized piece of natural mica.

### Surface characterization

The topography image of the surface in the main text and supplementary materials was measured using Asylum Research MFP-3D Infinity AFM in tapping mode (resolution: 256 × 256 pixels). After 100 km of sliding between the graphite and DLC, we took the disk from the turntable and placed it under AFM. According to the mark made in advance, we found the location of the sliding track. We characterized a 10 μm × 10 μm square area with the sliding track as the center, as shown in Fig. 1e. Because the size of the graphite flake was 4 μm × 4 μm, the 10 μm × 10 μm zone covered the entire sliding track.

If wear occured, there would be defects at the bottom surface of the graphite flake which was in contact with DLC. Raman spectra are widely used to characterize graphitic materials and particularly sensitive to the defects in graphite [53]. When 100 km of contact sliding was finished, we turned over the graphite flake sample and characterized it using Raman spectra, as shown in Fig. 1d and Supplementary Fig. S5. The absence of D peak shows there are no defects appearing on the graphite sample, thus no wear has occurred.

### Friction measurements

The normal force and lateral force applied by the AFM tip were calibrated by Sader's method [54] and a diamagnetic calibrator [55] respectively (Supplementary Section 10). Before the measurements, several hundred friction loops were conducted to remove surface contaminants by wiping effect [56] and diffusion effect [57] in the sliding direction. The frictional stress would reach a steady state in this process [58], followed by the frictional force measurement.

## Supplementary Material

nwab109_Supplemental_File
