# Supplementary material for: 100 km wear-free sliding achieved by microscale superlubric graphite/DLC heterojunctions under ambient conditions
Source: Natl Sci Rev. 2021 Jun 24;9(1):nwab109. doi: 10.1093/nsr/nwab109 (PMC8776547; doi:10.1093/nsr/nwab109)
Supplement: nwab109_Supplemental_File [file nwab109_supplemental_file.docx]

**Supplementary Materials**

This supplementary material contains the following contents,

1. Methods to obtain the friction coefficient

2. Thickness of the graphite flake on substrates

3. Robust superlubricity for graphite flake sliding on DLC, α-Al_2_O_3_, and silicon

4. Raman spectrum at other locations of the graphite flake after 100 km sliding on DLC

5. Estimation of the wear rate

6. Calibration of the normal force during 100 km sliding process

7. Details of the finite element methods

8. Theoretical analysis of the full contact condition

9. AFM characterization of the seven substrates

10. Calibration of normal force and Lateral force in AFM measurements

11. Discussion about the COF between the vdW layered materials and non-vdW layered materials.

12. Dependence of the measured friction on humidity for graphite/DLC heterostructure

1. **Methods to obtain the friction coefficient**

Figure S1 presents the friction loops between the graphite flakes and seven substrates. The closed area by the forward and backward curves represents the frictional energy dissipation. The average frictional stress varies from 0.015 MPa to 0.03 MPa for 40 μN normal load, comparable to the value of 0.03 MPa measured in superlubric graphite/hexagonal boron nitride heterojunctions [1] and 0.01 MPa measured in superlubric misaligned homogeneous graphitic contacts [2].


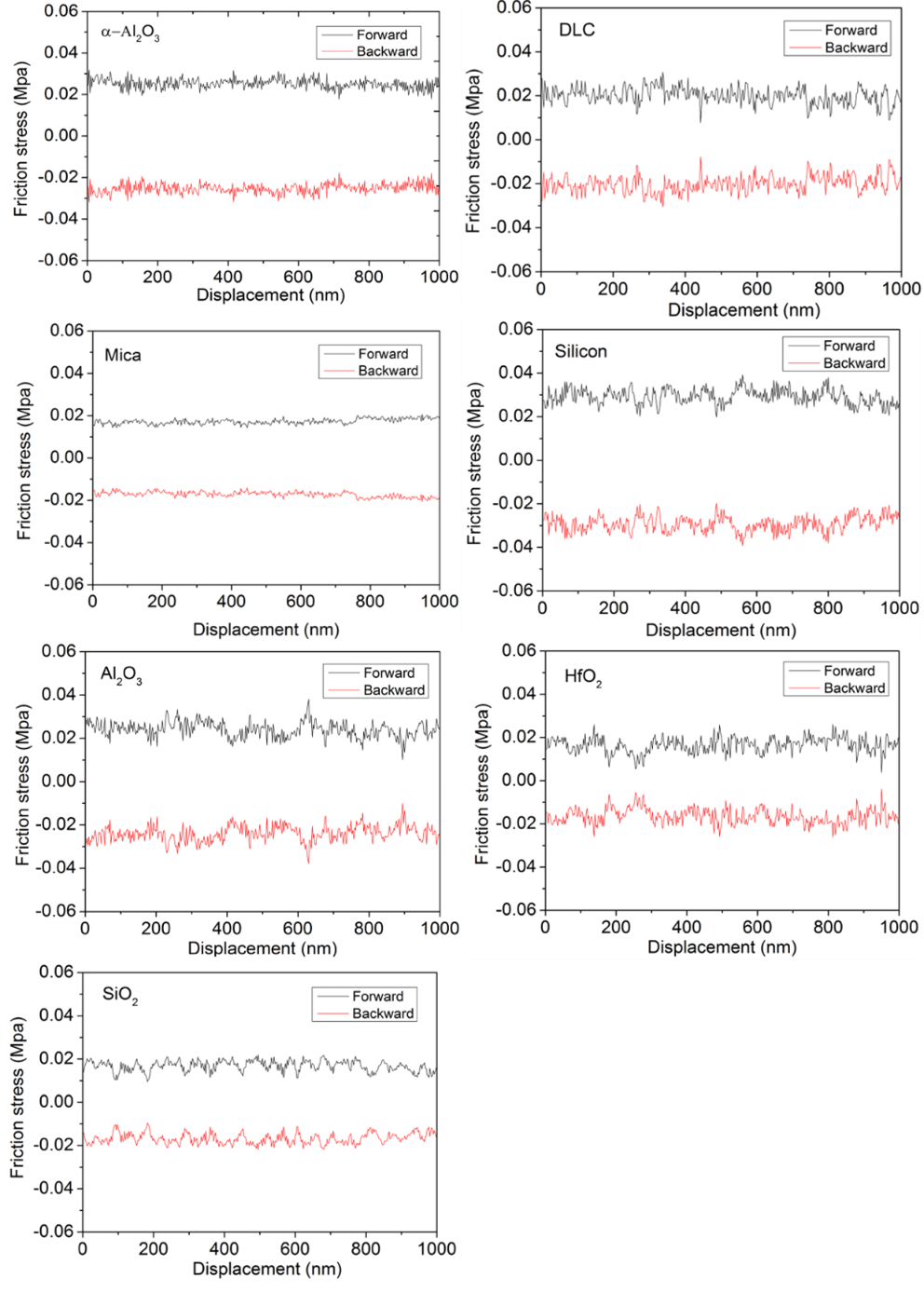


Figure S1. Friction loop between the graphite flakes and seven substrates, the normal force was about 40 μN and the scan velocity was kept constant at 1500 nm/s.

Figure S2 shows the typical dependence of friction on normal force between the graphite flakes and seven substrates. The average friction force increases linearly with normal load for all seven substrates. Notably, the friction seems not to be zero when normal force is zero because of the adhesion force on friction. Such phenomenon is also observed in a previous work [3], where a graphite-coated microsphere on SiO_2_ is slid on graphite substrates. The friction force can be written as *F*_f_ = *μF*_N_ + *F*_0_, where *F*_f_ is friction force, *μ* represents the COF, *F*_N_ is normal force and *F*_0_ is the offset friction force when *F*_N_=0. By fitting the slope between friction and normal force we obtained the COF. All of COFs of the seven materials fall on the order 10^−3^_,_ as shown in Fig. 2d in the main text.


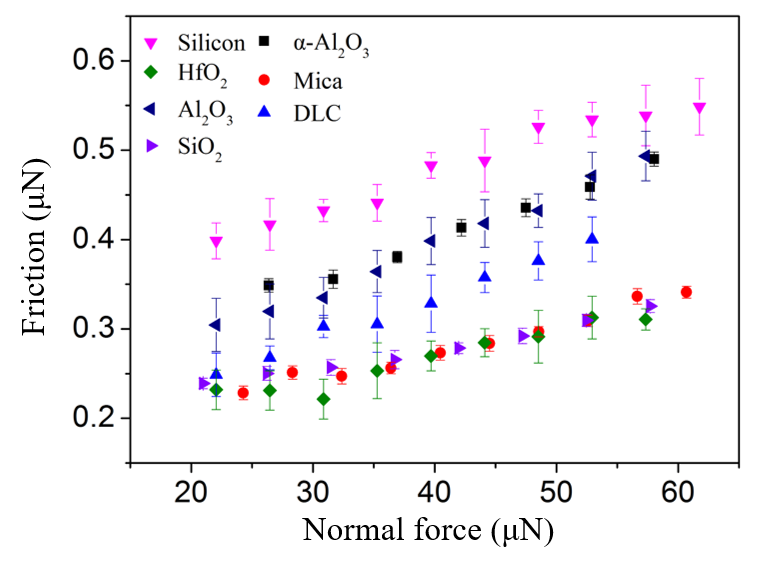


Figure S2. Measurements of the friction coefficient between the graphite flakes and seven substrates. During friction measurements the scan velocity was kept constant at 1500 nm/s. The error bars represent the standard deviation of the results obtained from 8 independent friction measurements.

1. **Thickness of the graphite flake on substrates**

Tapping-mode AFM was used to measure the thickness of the graphite flake sliding on the substrates. Figure S3a shows a typical height image of a graphite flake on the substrate. The square graphite flake is 4 μm in length, with a height of about 205 nm (100 nm thick cap, see Fig. S3b). We measured 10 flake’s thickness, which vary from 125 nm to 370 nm. The average height is about 207 nm, with a standard deviation of 66 nm.


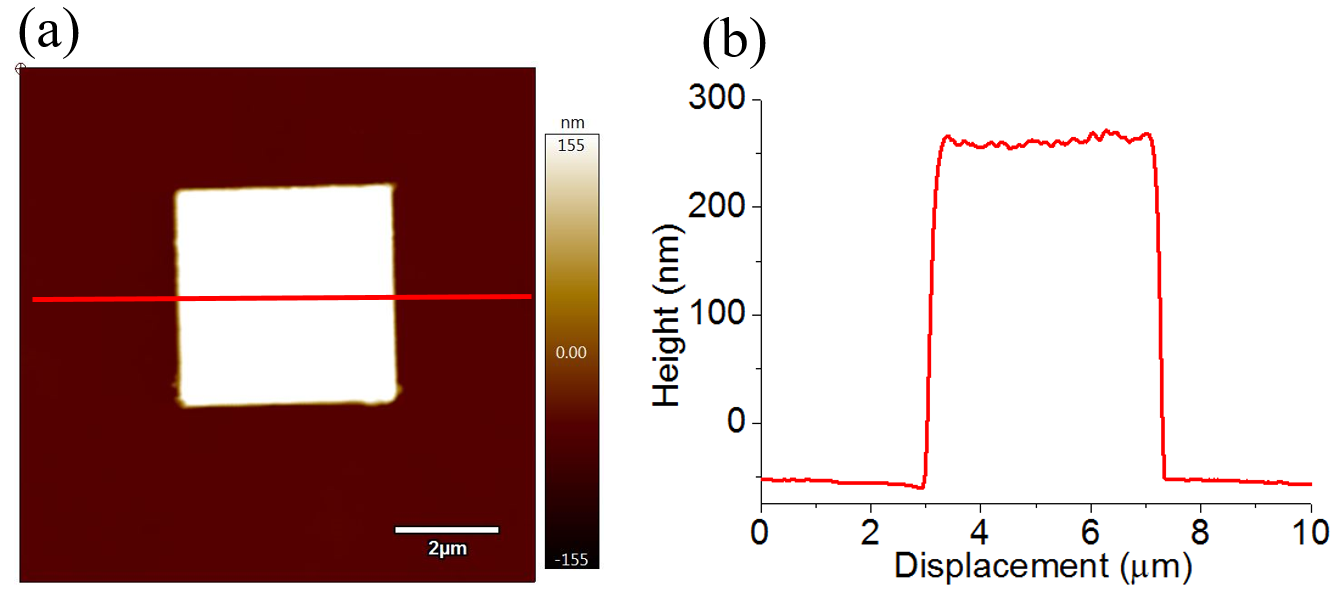


Figure S3. The height map of a graphite flake on the substrate. (a) Height map of a graphite flake. (b) The cross-sectional height map corresponding to the red line of (a).

1. **Robust superlubricity for graphite flakes sliding on DLC, α-Al_2_O_3_, and silicon**

The time evolution of friction reflects the stability of the friction system and whether wear occurs for the friction pair. As shown in Fig. S4, in the tested range of up to 1400 friction loops, the friction between the graphite flakes and the three substrates is very stable, shows no significant drift. This indicates that the presence of mechanical wear is effectively hindered in the systems by superlubric sliding.


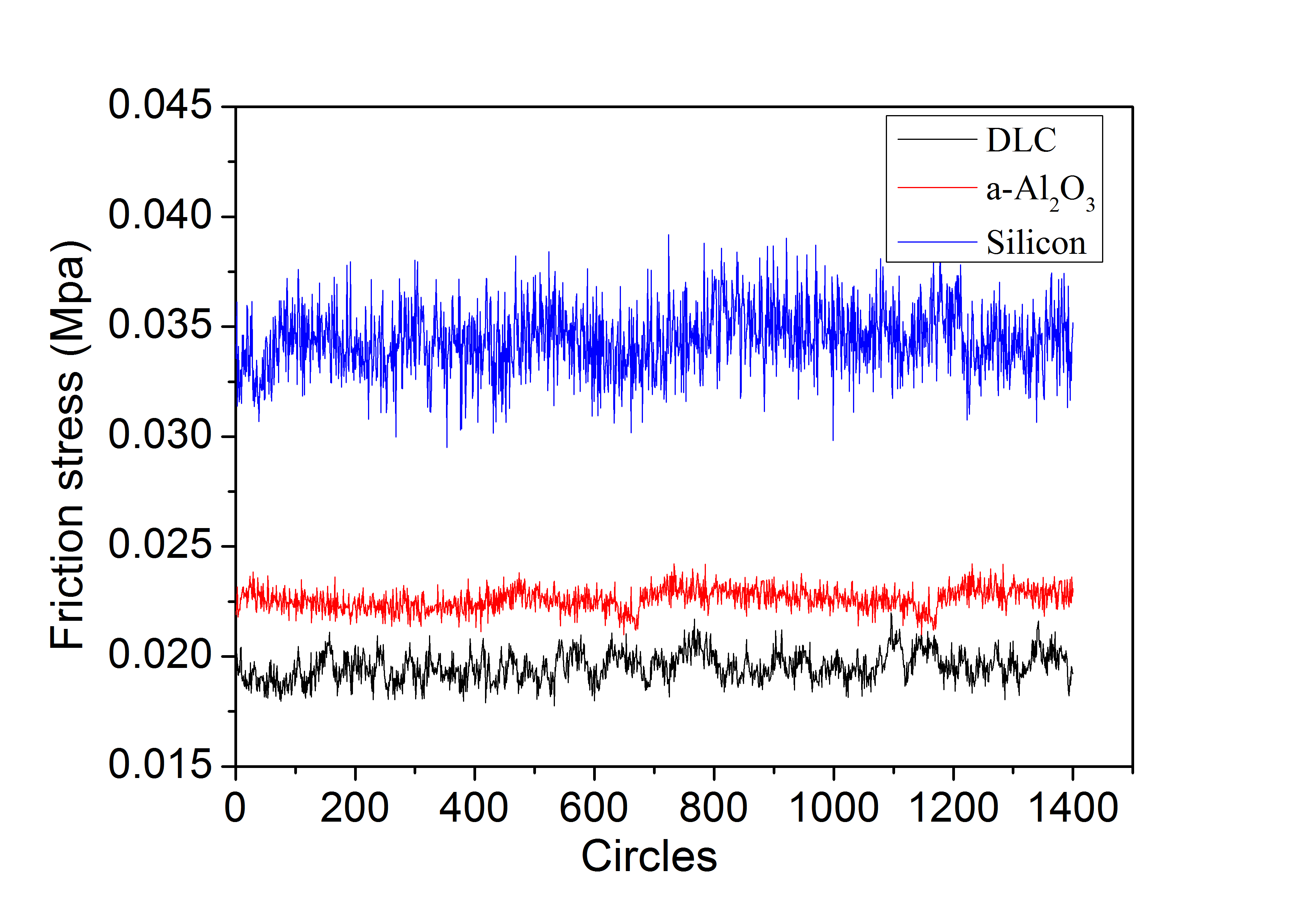


Figure S4. Time evolution of friction up to 1400 friction loops between the graphite flakes and three substrates: DLC, α-Al_2_O_3_ and Silicon, which were performed under ambient condition. The normal force was about 52 μN and scan velocity was kept at 2000 nm/s.

1. **Raman spectrum at other locations of the graphite flake after 100km sliding on DLC**

In Figs. 1d-e of the main text, we presented the optical image and the central point’s Raman spectrum of the graphite flake’s surface which was in contact with the DLC substrate during the 100 km sliding. The laser size of our Raman is about 1 μm. To confirm that there is no wear on other areas of the flake, we picked another four points around the corners for characterizations (Fig. S5a). All the spectra are free of D peak, indicating no wear.


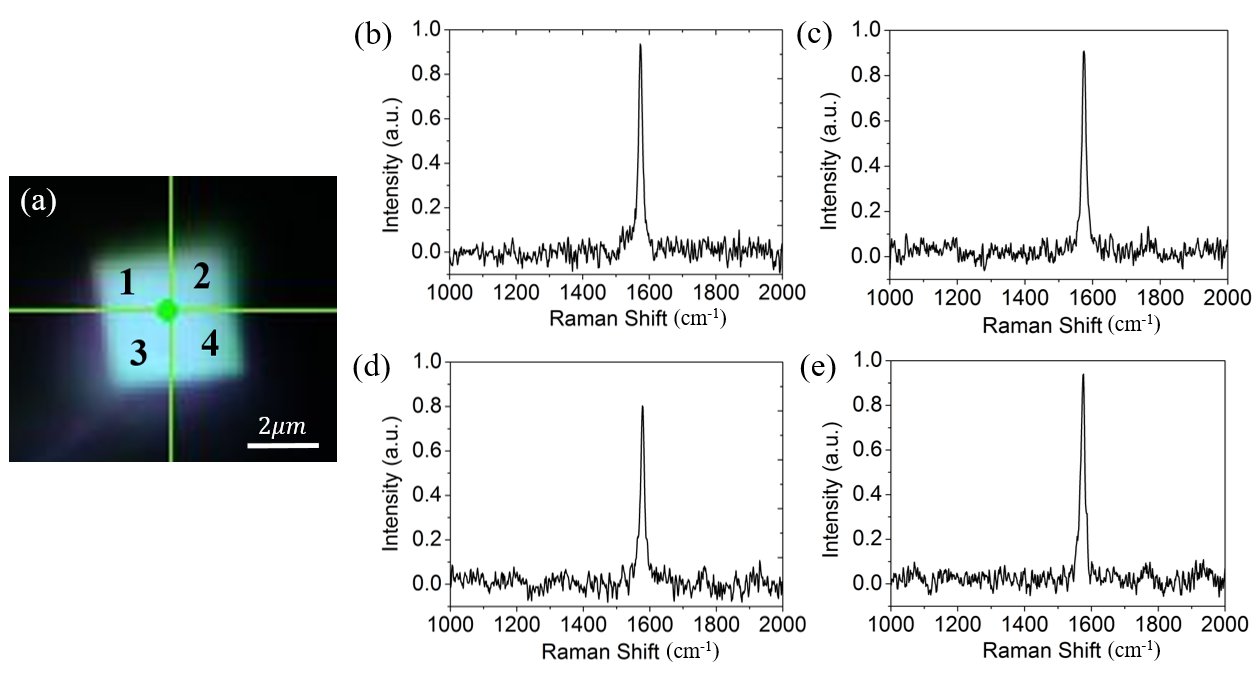


Figure S5. Raman spectra of other four points within the graphite flake after 100 km sliding. (a) The same image of Fig. 1c with 4 marks. (b-e) Raman spectrum measured at point 1-4 respectively.

1. **Estimation of the wear rate**

As the microscale graphite flake was glued to the probe and could hardly be moved to a substrate without damage, we had to take this thin probe together to do characterizations, which brings more noise. Although we find no D peak in Fig. 1d in the main text, it may be that the D peak is masked in the noise. The concentration of vacancy *C*_v_ can be obtained by the Raman intensity ration *I*_1355_/*I*_1580_, as follows [4]:

*C*_v_=*k*·(*I*_1355_/*I*_1580_)^2^,

where *k* is a constant. *I*_1355_ and *I*_1580_ are the intensities of the peaks at 1355 cm^−1^ and 1580 cm^−1^.

Considering the amplitude of the noise in Raman spectrum in Fig. S5, we estimate the biggest value of the Raman intensity ratio *I*_1355_/*I*_1580_ to be 0.1. Taking *k* as 0.022 [4], the concentration of vacancy *C*_v_ can be estimated to be 0.00022. For the normal force being 10 μN, the sliding distance being 100 km, and the average thickness of the graphite flake being 207 nm, the wear rate is estimated to be smaller than 7.2×10^−13^ mm^3^/N·m, which is negligible.

1. **Calibration of the normal force during 100 km sliding process**

When loading the graphite flake with the tungsten probe, the probe was bent, and the flake slid forward, as sketched in Figs. S6b-d. During the unloading stage, such process was reversed. Figure S6a shows the set-up of the calibration experiment. The resolution of the balance is 0.01 mg, approximately 0.1μN (for *g*≈10 m/s^2^). For each micrometer during the flake sliding either forward upon loading or backward when unloading, the normal force was recorded. The obtained calibration result is shown in Fig. S7. As the fake slides forward, the normal force increases linearly. The loading curve and unloading curve coincide with each very well, being consistent with the small deformation theory of the beam. Based on the calibration result, by monitoring the position of the flake during the 100 km slide on DLC, we estimate the normal force applied on the graphite flake to be about 10 μN.


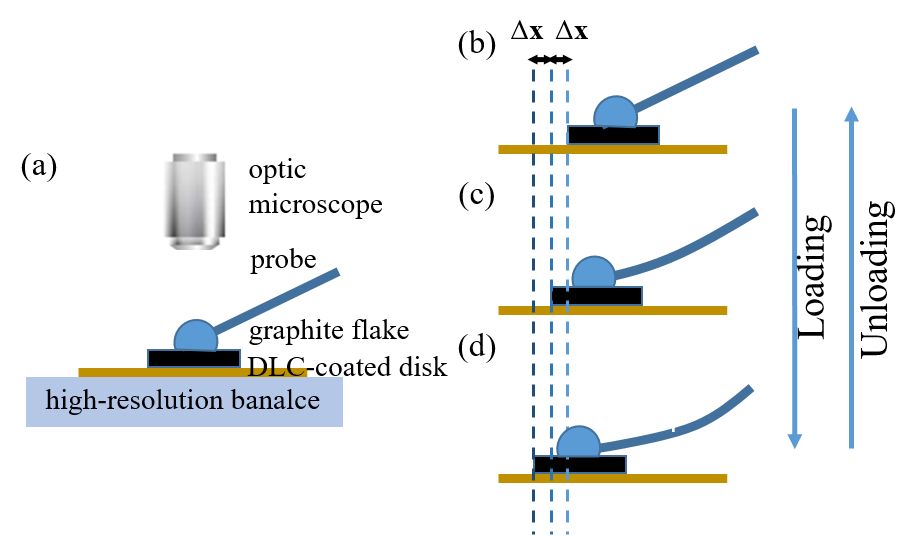


Figure S6. Calibration method of normal force applied by the tungsten probe. (a) Schematic diagram of the calibration equipment. (b) No normal force applied by the probe. (c) Load the probe to move the flake forward by Δ*x*. (d) Larger normal load leads to further movement of the flake. During the unloading process, such process is reversed.


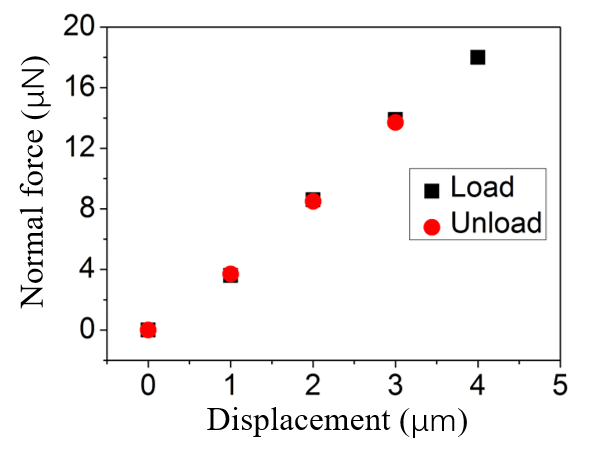


Figure S7. Calibration results. The horizontal axis of displacement corresponds to Δ*x* in Fig. S6, where Δ*x*=1μm.

1. **Details of the finite element methods**

The cross-sectional geometries of the FEM model are consistent with the graphite mesa sample and the substrate, which is shown in Fig. 3c. The thickness of Au and Al are both 50 nm, which consist the cap of the mesa, and graphite’s thickness is 100 nm (the thinnest is 125 nm in our experiments). We simplified the shape of the cross-sectional of the substrate into a sinusoid. The maximum peak-to-peak value is 1.4 nm with a wavelength of 60 nm. The Young's Modulus and Poisson's ratio of Au and Al are 79 GPa and 0.42, 70GPa and 0.35, respectively [5]. The elastic parameters of the graphite are given through the following stress-strain relation [6-8]:

$$\left\{ \begin{aligned} \sigma_{x} \\ \sigma_{z} \\ \tau_{\text{xz}} \end{aligned} \right\}=\left[ \begin{matrix} 1060 & 180 & 0 \\ 180 & 1060 & 0 \\ 0 & 0 & 0.4 \end{matrix} \right]\left\{ \begin{aligned} \varepsilon_{x} \\ \varepsilon_{z} \\ \gamma_{\text{xz}} \end{aligned} \right\}$$

where the elastic modulus is given in units of GPa. Given that the rigidity of the substrates is much greater than graphite and its metal cap, we take the substrate as a rigid body. The interfaces of Al/Au and Au/graphite are both bonded. The interaction of the graphite/substrates interface is described by the curve in Fig. S8 to mimic the vdW interaction, which is derived from Lennard-Jones (LJ) potential and will be discussed later. The substrate is fixed. The simulating results is shown in Fig. 3d in the main text, which clearly show that the adhesive force (924 MPa) can overcome the de-adhesion force (< 400 MPa) caused by the bending of the graphite mesa when the graphite’s thickness is 100 nm.


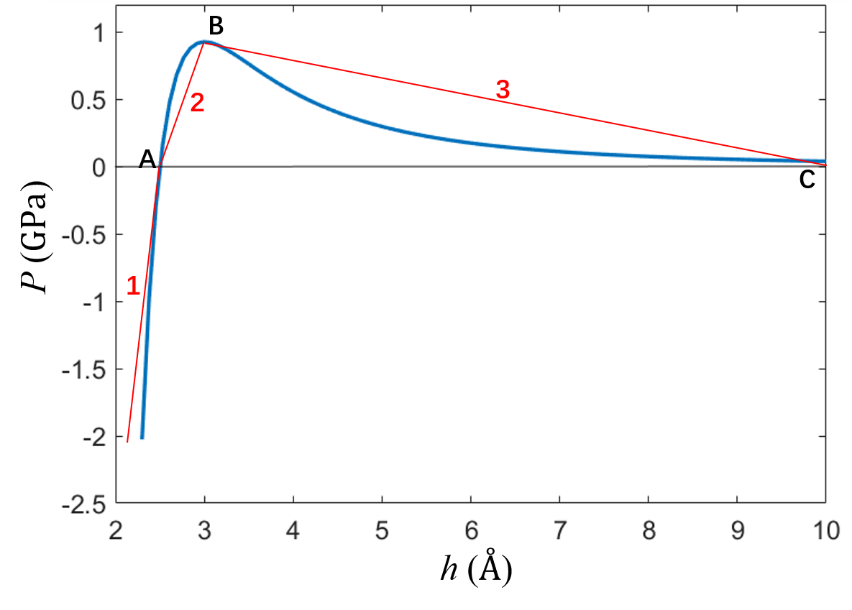


Figure S8. Interactions between graphite and substrate. Blue line is the vdW interaction derived by LJ potential between carbon atoms. Red line is the simplified interactions used in the FEM methods, which consists three straight lines. Line 1 is the tangent of the blue curve at point A. Line 2 connects point A and point B, and line 3 connects point B and point C.

The vdW interaction exists between the bottom surface of the graphite and the top surfaces of the substrate. The constitutive pressure-displacement relation is derived from the LJ potentials that are widely used to describe the vdW interactions. The LJ potential between two carbon atoms is given by [9]:

$$\begin{aligned} V_{ij}=4\varepsilon\left[ \left( \frac{\sigma}{r_{ij}} \right)^{12}-\left( \frac{\sigma}{r_{ij}} \right)^{6} \right], \end{aligned}$$

where $\sigma=3.5Å$, and $\varepsilon=0.07 kcal/mol$. The interatomic distance $r_{ij}$ is

$$\begin{aligned} r_{ij}=\sqrt{r^{2}+\left( z_{1}-z_{0} \right)^{2}}, \end{aligned}$$

where *r* is the in-plane distance, *z*_1_ and *z*_0_ is the z-axis coordinate of the upper and lower surface atom respectively. The total potential between these two flat interfaces is

$$V_{tot}\left( h \right)=\rho_{1}\rho_{2}\int_{-\infty}^{0} \int_{h}^{\infty} \int_{0}^{\infty} V_{ij}2\pi rdrdzdz_{0}$$

$$\begin{aligned} =4\varepsilon\rho_{1}\rho_{2}\pi\left( \frac{\sigma^{12}}{360h^{8}}-\frac{\sigma^{6}}{12h^{2}} \right), \end{aligned}$$

where $\rho_{1}\approx1.76\times{10}^{29} m^{-3}$ and $\rho_{2}\approx1.14\times{10}^{29} m^{-3}$ is the atom number density of the upper and lower surfaces, *h* is the distance between two surfaces.

The pressure between these two flat interfaces $P(h)$ is

$$P\left( h \right)=-\frac{{dV}_{\mathrm{tot}}\left( h \right)}{dh}=\varepsilon\rho_{1}\rho_{2}\pi\left( \frac{4\sigma^{12}}{45h^{9}}-\frac{2\sigma^{6}}{3h^{3}} \right),$$

For $P\left( h \right)=0$, $h_{0}=\sqrt[6]{\frac{2}{15}}\sigma=0.25 nm$, which corresponds to point A in Fig. S8, and the $V_{tot}$ at the balance position is $V_{m}=V_{tot}\left( h{}_{0} \right)=-0.2253 J/m^{2}$.

To calculate the maximum adhesive pressure, let $\frac{dP(h)}{dh}=0$, $h_{1}=\sqrt[6]{\frac{2}{5}}\sigma=0.30 nm$, which corresponds to point B in Fig. S8. The maximum adhesive pressure $P_{m}=P\left( h_{1} \right)=-0.924 GPa$. For *h* being larger than 1 nm, the vdW interaction is negligibly small, thus considering to be zero (point C in Fig. S8).

1. **Theoretical analysis of the full contact condition.**

To get quantitative understanding on the contact condition, we proposed the following model. In our experiments, the graphite flake is capped with the rigid metal layer which sustains the external normal load $p_{zz}$ (Fig. S9). By applying reasonable assumptions as shown below, we define the total potential function $\Phi$ by considering the competence between the work done by external pressure *W*, the work done by the interfacial adhesion $\Gamma$, and the elastic energy of the graphite flakes *U*.


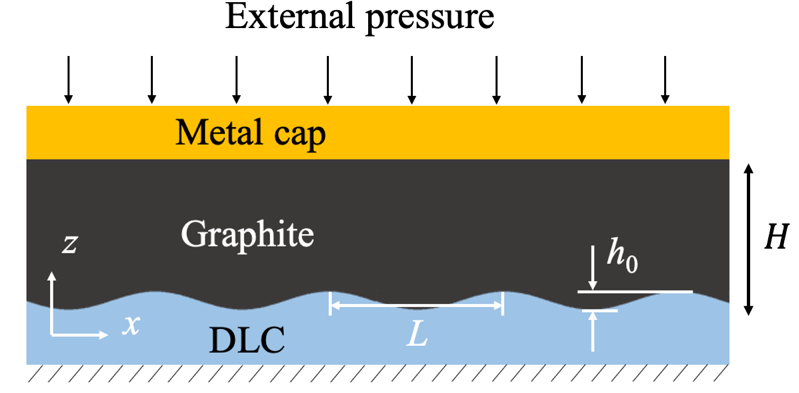


Figure S9. Schematic diagram of the contact condition.

*Assumption* 1. The elastic energy is mainly contributed by the component $\sigma_{zz}\varepsilon_{zz}$.

The rationality of this assumption is supported by the existing experiments and simulations. Since graphite is a transversely isotropic vdW layered material, with its inter-layer stiffness (on the order of kPa~MPa [8,10]) much smaller than the intra-layer stiffness (~1 TPa [11]), it can slide between layers easily. This indicates that the elastic energy contributed by the interlayer shear stress/strain is comparably negligible in the graphite flake. Recent experiments studying the bending stiffness of graphite also observed that the interlayer shear stress is negligible in graphite [12,13].

*Assumption* 2. The small deformation assumption.

The height and lateral width of the corrugation is *h*_0_≤1.4 nm, and *L*=60 nm, which gives *h*_0_/*L*~0.01. This assumption linearizes the interaction and deformation in the following discussion.

For a simplified 2-Dimensional model (Fig. S9), the total potential energy $\Phi$ can be given by,

$$\begin{aligned} \Phi=U-\Gamma-W,\#\left( S1 \right) \end{aligned}$$

where *U* is the elastic energy, $\Gamma$ is the work done by the interfacial adhesion, and *W* is the work done by the external load. By further assuming the cross section of the rough peak to be a sinusoidal shape,

$$\begin{aligned} h\left( x \right)=h_{0}\sin^{2} \left( \frac{\pi}{L}x \right),\#\left( S2 \right) \end{aligned}$$

where *h*_0_=1.4 nm and *L*=60 nm is the height and lateral period of the corrugation, the elastic energy of the graphite flake can be given by

$$\begin{aligned} U=\frac{a}{L}\int_{-L/2}^{L/2} \frac{1}{2}E\varepsilon_{zz}^{2}\left[ H-h\left( x \right) \right]dx,\#\#\left( S3 \right) \end{aligned}$$

where $a=4 \mu m$ is the side length of the graphite mesa, *E*=36.5 GPa is the elastic constant, *H* is the total height of the graphite flake, $\varepsilon_{zz}=h\left( x \right)/H$ is the *zz* component of the strain tensor. Thus, Eq. S3 can be simplified to

$$\begin{aligned} U=\frac{3Eah_{0}^{2}}{16H},\#\left( S4 \right) \end{aligned}$$

By substituting the adhesion work

$$\begin{aligned} \Gamma=a\gamma,\#\left( S5 \right) \end{aligned}$$

and external work

$$\begin{aligned} W=p_{zz}ah_{0},\#\left( S6 \right) \end{aligned}$$

into

$$\begin{aligned} \Phi=U-\Gamma-W\leq0,\#\left( S7 \right) \end{aligned}$$

one can finally get

$$\begin{aligned} H\geq\frac{3Eah_{0}^{2}}{16\left( \gamma a+p_{zz}h_{0}a \right)}\approx59.2 nm.\#\left( S8 \right) \end{aligned}$$

Here, according to our experiments, we use $p_{zz}=1 \mathrm{MPa}$ and the surface energy, $\gamma=0.2253 J/m$ as estimated in section 7. Therefore, with Eq. S8, the lower limit of *H* is 59.2 nm, this value decreases with the increase of the external pressure $p_{zz}$, and *H*=60 nm when $p_{zz}=0$ (Fig. 3b in the main text). Since *H* in our experiments ranges from 125 to 370 nm, it is reasonable to assume a full contact at the interface between graphite flake and the substrates.

In the above discussion, we focus on the total potential energy of the full-contacted state, i.e., $\max\left\{ h\left( x \right) \right\}=h_{0}$. However, the full-contacted state only represents the end of a “successful” contact process. To achieve this “successful” process, two other questions should also be considered:

1. Is there any energy barrier during the contact process?
2. Is the contact state stable?

In the contact process, the graphene at the contact interface tends to shrink to fit the corrugated substrate. At the same time, the friction impedes this contraction. Therefore, the friction could introduce the energy barrier during the contact process.

However, thanks to the superlubric state of the interfaces as discussed in the main text, the shear strength should be much smaller than the interlayer shear strength of graphite, thus being negligible in our discussion.

Besides, instead of considering a full-contact state, let us assume a partially contacted state with a revised Eq. S2 where the amplitude of the lower surface of graphite $h_{g}\leq h_{0}$ is a variable,

$$\begin{aligned} h\left( x \right)=h_{g}\sin^{2} \left( \frac{\pi}{L}x \right),\#\left( S9 \right) \end{aligned}$$

The elastic energy, the work done by the interfacial adhesion and the work done by the external loads can be rewritten as,

$$\begin{aligned} \left\{ \begin{aligned} U=\frac{3Eah_{g}^{2}}{16H} \\ \Gamma=a\gamma h_{g}/h_{0} \\ W=p_{zz}ah \end{aligned} \right. , h_{g}\in\left[ 0, h_{0} \right].\#\left( S10 \right) \end{aligned}$$

The total potential is

$$\begin{aligned} \Phi=\frac{3Ea}{16H}h_{g}^{2}-\left( \frac{a\gamma}{h_{0}}+p_{zz}a \right)h_{g},\#\left( S11 \right) \end{aligned}$$

The stable state is achieved when

$$\begin{aligned} \frac{d\Phi}{dh_{g}}=\frac{3Ea}{8H}h_{g}-\left( \frac{a\gamma}{h_{0}}+p_{zz}a \right)=0,\#\left( S12 \right) \end{aligned}$$

Eq. S12 gives *h_g_* corresponding to the stable configuration

$$\begin{aligned} h_{stable}=\frac{8H}{3E}\left( \frac{\gamma}{h_{0}}+p_{zz} \right).\#\left( S13 \right) \end{aligned}$$

In our experiments, *H*>125 nm, we get $h_{stable}>1.48 nm>h_{0}.$ Above all, we believe the full contact state for our system is stable and achievable in experiments.

1. **AFM characterization of the seven substrates**
2. Sapphire, Ra: 40.594 pm


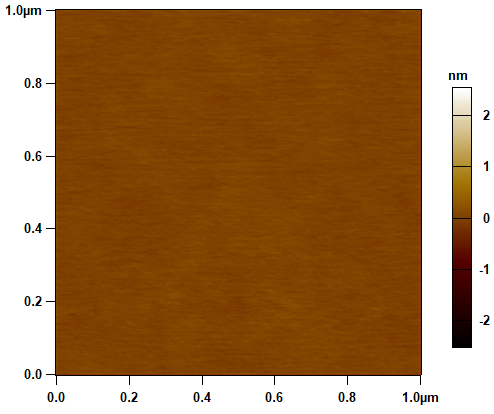


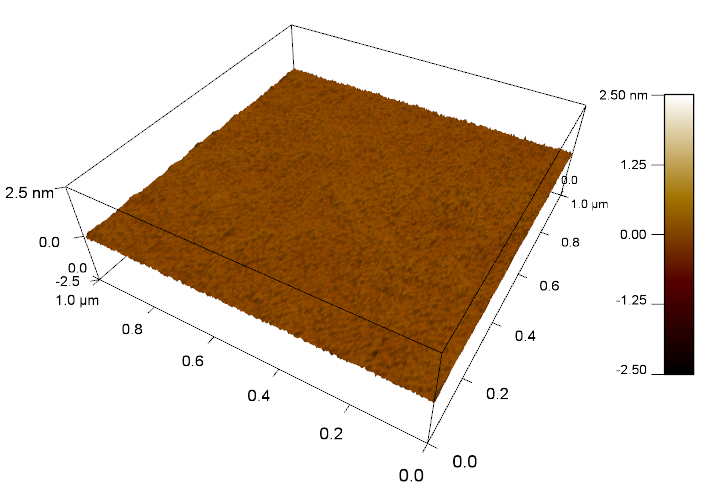


1. Mica, Ra: 88.667 pm


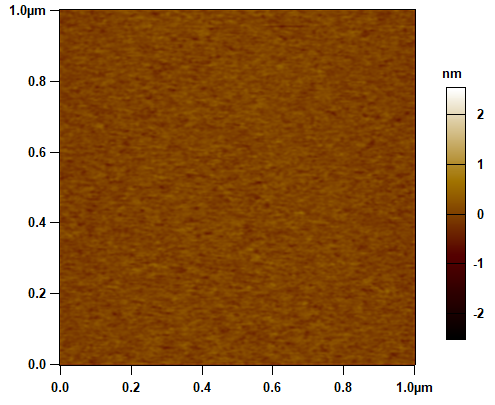


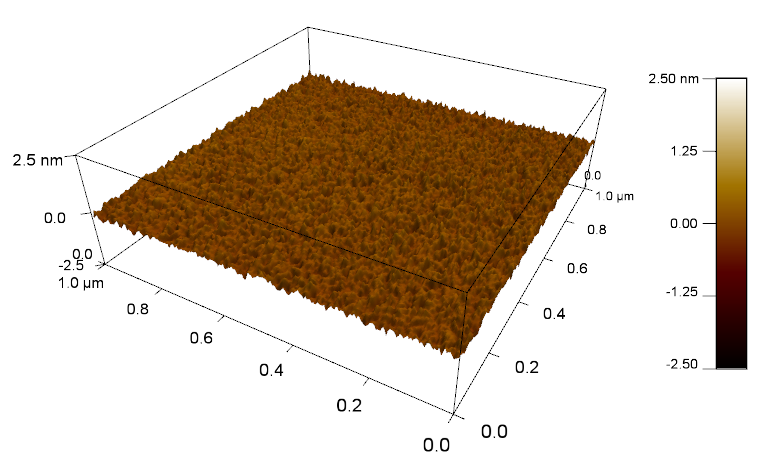


1. DLC, Ra: 91.110 pm


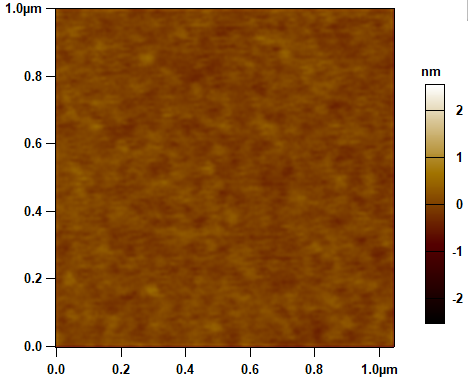


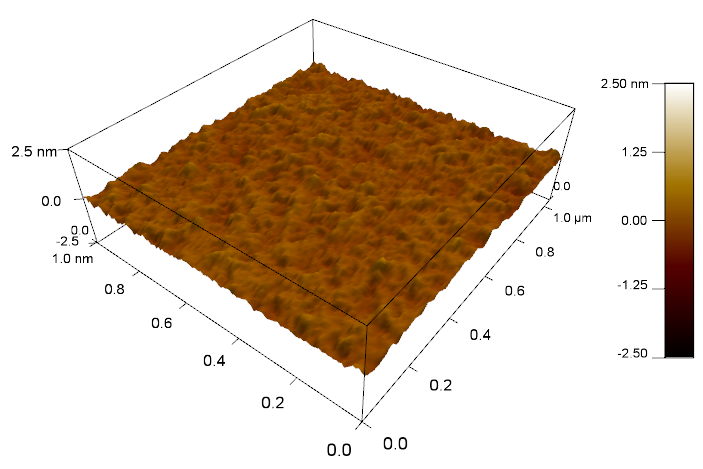


1. Silicon, Ra: 161.098 pm


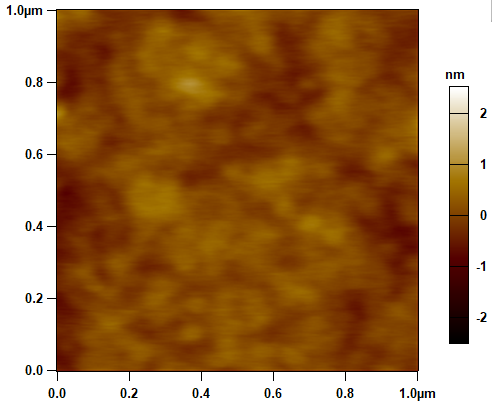


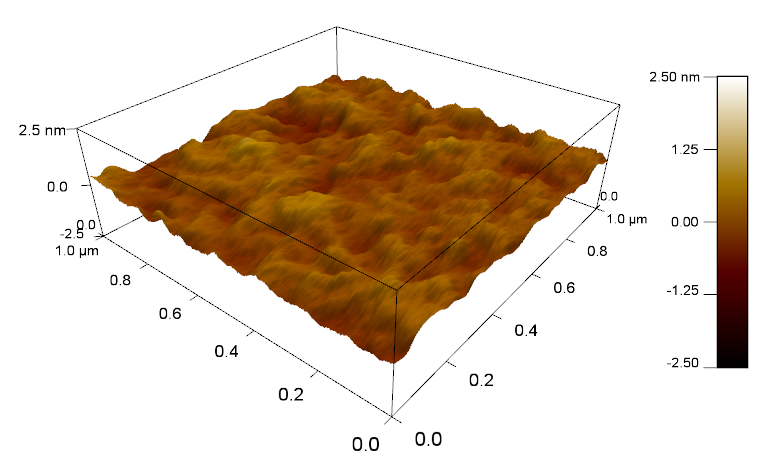


1. HfO_2_, Ra: 229.088 pm


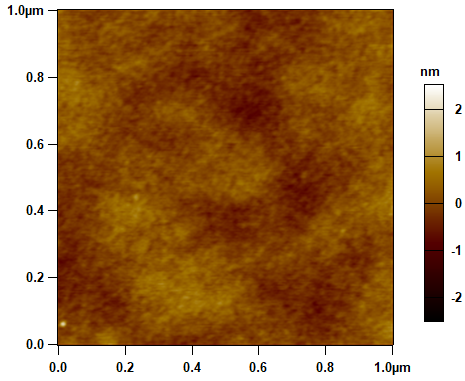


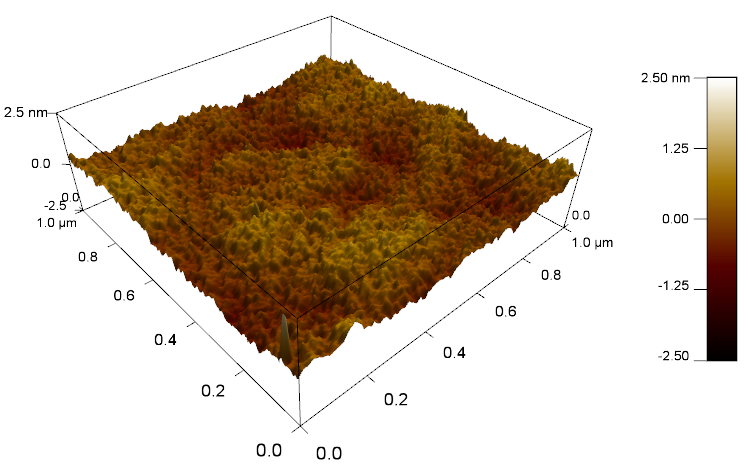


1. Al_2_O_3_, Ra: 285.094 pm


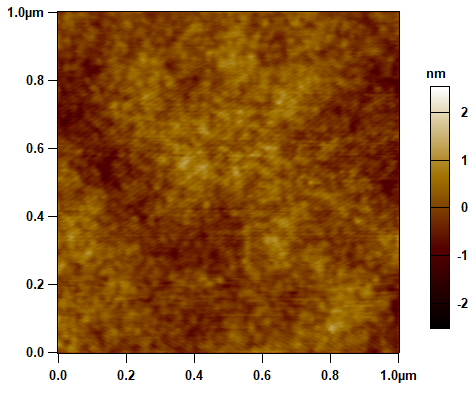


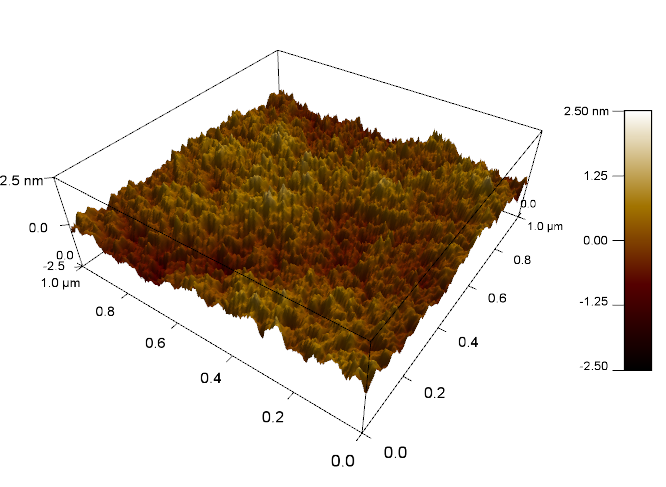


1. SiO_2_, Ra: 339.663 pm


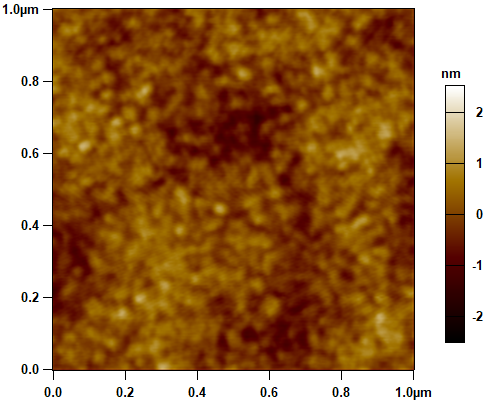


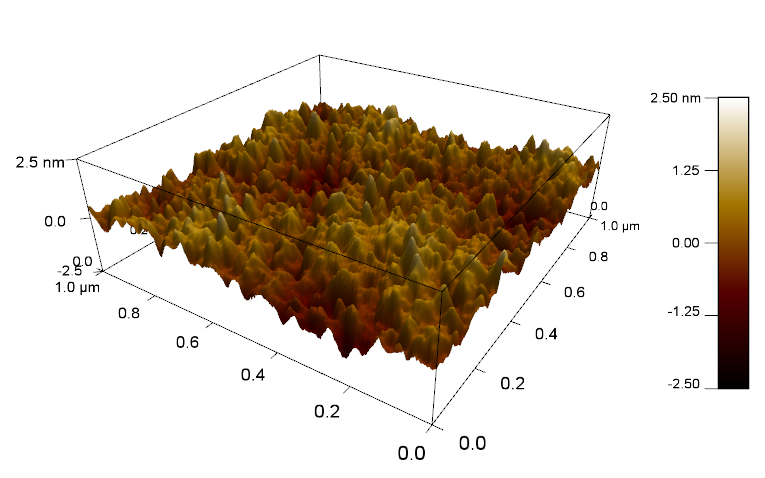


**10. Calibration of normal force and Lateral force in AFM measurements**

The Sader method [14] was used to calibrate the normal force. Using built-in subroutine of AFM control software, the Sader stiffness 𝑘_sd_ of the AFM cantilever can be estimated. Then, make the AFM tip press on a hard substrate to obtain the electrical signal of normal deflection (DFL) v.s. displacement in normal direction curve. The optical sensitivity factor in normal direction 𝑘_n-slope_ can be obtained by fitting the curve linearly. Finally, the normal force (F*_N_*) that AFM tip applied can be obtained as 𝐹_𝑁_ = DFL × 𝑘_sd_/𝑘_n-slope_. For example, in an experimental measurement, the 𝑘_sd_ is measured to be 50 N/m, and the 𝑘_n-slope_ is measured to be 9.48 nA/μm. When DFL is controlled to be 10 nA, the 𝐹_𝑁_ is 52.76 μN.

The lateral force calibration was performed using a diamagnetic lateral force calibrator (D-LFC) [15] in which a square sheet of pyrolytic graphite is levitated above four NdFeB magnets. Make the AFM tip press on the suspended graphite and do the lateral reciprocating motion to obtain the electrical signal of lateral deflection (LF) v.s. displacement *x* in lateral direction curve. The optical sensitivity factor in lateral direction 𝑘_l-slope_ can be obtained by fitting the curve linearly. The lateral displacement *x* of the pyrolytic graphite is proportional to the shear force *F_f_* acting upon it, that is, *F_f_ =k_d_*·*x*, where *k_d_* is the D-LFC spring constant which can be measured before the calibration process [15]. Finally, the lateral force (F*_f_*) that AFM tip applied can be obtained as 𝐹_f_ = LF × 𝑘_d_/𝑘_l-slope_. For example, in an experimental measurement, the 𝑘_d_ is measured to be 0.0305 N/m, and the 𝑘_l-slope_ is measured to be 1.814 pA/μm. When the value of LF is measured to be 0.02 pA, the corresponding lateral force F*_f_* is 0.34 μN.

**11. Discussion about the COF between the vdW layered materials and non-vdW layered materials.**

We noticed that compared with graphite/graphite homojunction [16,17] and graphite/hBN heterojunction [1], the COF measured here is about ten times larger (2×10^−3^ to 6×10^−3^ shown in Fig.1b compared with <1.4×10^−4^ [1,16,17]). An obvious difference between these two categories of systems is the larger roughness studied here. As illustrated in Fig. 3c and characterized in SM, there are mild asperities on the substrates with their maximum height being about 1.4 nm and lateral dimension about 60 nm. During the frictional test, when the graphite flake is sliding on the substrates, due to the close contact between the surfaces, intuitively the asperities on the substrates will induce deformation within the graphite counter-surface. Since the elastic modulus normal to the basal plane of the graphite is relatively low, the apparent indentation depth within the graphite flake will be comparable to the height of the asperity. To accommodate the deformation, the range of strain field within the graphite flake would be order larger. Zooming in at the interface between one asperity and the graphite surface, their sliding process is similar to a typical nanotribological test where the AFM tip is sliding on graphite, only being upside-down. For AFM tip sliding on graphite surfaces, there have been many studies [18]. A common belief is that besides the friction at the interface between the tip and the top layer of graphite, the deformation within the substrate caused by the tip sliding will also cause additional energy dissipation [19-21]. On microscale, when an asperity scratches the surface, the overall friction could also be regarded as the sum of sliding friction and ploughing friction [22]. During the loading process, both COFs are positive [23]. Thus, the overall COFs measured for graphite flake sliding on the surface with nanoscale asperities would be larger than that between atomically smooth 2D materials junctions [24].

**12. Dependence of the measured friction on humidity for Graphite/DLC heterostructure**

As superlubric system of DLC/DLC are sensitive to humidity, it is necessary to check the condition for our Graphite/DLC superlubric system. We tested the dependence of the measured frictional stress on the sliding velocity both under ambient condition and nitrogen atmosphere for comparative studies. The results are shown in Fig. S10. It is evident that the humidity plays a minor role in friction. In fact, such phenomenon has already been observed in other graphite based superlubric systems, such as graphite/hBN heterostructure [1] where humidity plays little role in friction. Another paper published recently also found that friction hardly changes with humidity for microscale superlubric graphite/gold heterostructure [25].


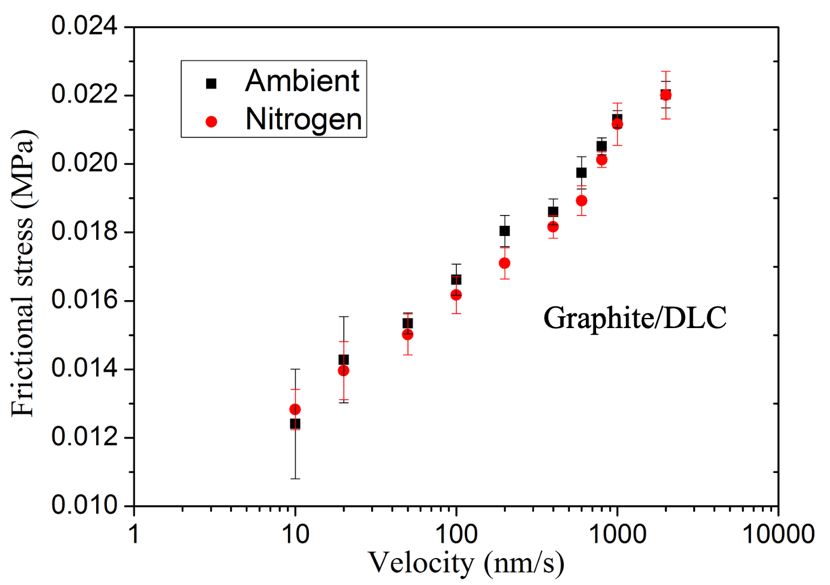


Figure S10. Dependence of the measured frictional stress on the sliding velocity. Measurements are performed under ambient conditions and nitrogen atmosphere. The load is about 50 μN. Temperature: 20~25 ℃.

**References**

1 Song, Y. *et al.* Robust microscale superlubricity in graphite/hexagonal boron nitride layered heterojunctions. *Nat. Mater.* **17**, 894-899 (2018).

2 Koren, E., Loertscher, E., Rawlings, C., Knoll, A. W. & Duerig, U. Adhesion and friction in mesoscopic graphite contacts. *Science* **348**, 679-683 (2015).

3 Liu, S.-W. *et al.* Robust microscale superlubricity under high contact pressure enabled by graphene-coated microsphere. *Nat. Commun.* **8**, 1-8 (2017).

4. Niwase, K. *Int. J. Spectrosc.* **2012**, 197609 (2012).

5 Martienssen, W. & Warlimont, H. Springer handbook of condensed matter and materials data (Springer Science & Business Media, 2006).

6 Kelly, B. T. Physics of graphite. (Applied science, 1981).

7 Bosak, A., Krisch, M., Mohr, M., Maultzsch, J. & Thomsen, C. Elasticity of single-crystalline graphite: Inelastic x-ray scattering study. *Phys. Rev. B* **75**, 153408 (2007).

8 Liu, Y., Xu, Z. & Zheng, Q. The interlayer shear effect on graphene multilayer resonators. *J. Mech. Phys. Solids* **59**, 1613-1622 (2011).

9 Sinclair, R. C., Suter, J. L. & Coveney, P. V. Graphene–graphene interactions: friction, superlubricity, and exfoliation. *Adv. Mater.* **30**, 1705791 (2018).

10 Wang, G. *et al.* Measuring Interlayer Shear Stress in Bilayer Graphene. *Phys. Rev. Lett.* **119**, 036101 (2017).

11 Lee, C., Wei, X. D., Kysar, J. W. & Hone, J. Measurement of the elastic properties and intrinsic strength of monolayer graphene. *Science* **321**, 385-388 (2008).

12 Wang, G. R. *et al.* Bending of Multilayer van der Waals Materials. *Phys. Rev. Lett.* **123**, 116101 (2019).

13 Han, E. *et al.* Ultrasoft slip-mediated bending in few-layer graphene. *Nat. Mater.* **19**, 305-309 (2020).

14 Sader, J. E., Chon, J. W. M. & Mulvaney, P. Calibration of rectangular atomic force microscope cantilevers. *Rev. Sci. Instrum.* **70**, 3967–3969 (1999).

15 Li, Q., Kim, K. S. & Rydberg, A. Lateral force calibration of an atomic force microscope with a diamagnetic levitation spring system. *Rev. Sci. Instrum.* **77**, 065105 (2006).

16 Vu, C. C. et al. Observation of normal-force-independent superlubricity in mesoscopic graphite contacts. *PRB* **94**, 081405(R) (2016).

17 Liu, Z. et al. Observation of Microscale Superlubricity in Graphite. *PRL* **108**, 5 (2012).

18 Zhang, S., Ma, T., Erdemir, A. & Li, Q. Tribology of two-dimensional materials: From mechanisms to modulating strategies. *Mater. Today* **26**, 67-86 (2019).

19 Mishra, M., Egberts, P., Bennewitz, R. & Szlufarska, I. Friction model for single-asperity elastic-plastic contacts. *Phys. Rev. B* **86**, 045452 (2012).

20 Smolyanitsky, A., Zhu, S., Deng, Z., Li, T. & Cannara, R. J. Effects of surface compliance and relaxation on the frictional properties of lamellar materials. *Rsc Advances* **4**, 26721-26728 (2014).

21 Sun, X. Y., Qi, Y. Z., Ouyang, W. G., Feng, X. Q. & Li, Q. Y. Energy corrugation in atomic-scale friction on graphite revisited by molecular dynamics simulations. *ACTA MECH SINICA* **32**, 604-610 (2016).

22 Lafaye, S., Gauthier, C. & Schirrer, R. The ploughing friction: analytical model with elastic recovery for a conical tip with a blunted spherical extremity. *Tribology Letters* **21**, 95-99 (2006).

23 Deng, Z., Smolyanitsky, A., Li, Q., Feng, X.-Q. & Cannara, R. J. Adhesion-dependent negative friction coefficient on chemically modified graphite at the nanoscale. *Nat. Mater.* **11**, 1032-1037 (2012).

24 Song, Y., Qu, C., Ma, M. & Zheng, Q. Structural Superlubricity Based on Crystalline Materials. *Small*, 1903018 (2019).

25 Li, J., Li, J., Chen, X., Liu, Y., & Luo, J. Microscale superlubricity at multiple gold–graphite heterointerfaces under ambient conditions. *Carbon* **161**, 827-833(2020).
